# Black-White Differences in The Effects of Parental Education on College Students’ Beliefs about Racial Discrimination

**DOI:** 10.31586/ojer.2024.997

**Published:** 2024-07-31

**Authors:** Shervin Assari, Hossein Zare

**Affiliations:** 1 Department of Internal Medicine, Charles R. Drew University of Medicine and Science, Los Angeles, CA, United States; 2 Department of Family Medicine, Charles R. Drew University of Medicine and Science, Los Angeles, CA, United States; 3 Department of Urban Public Health, Charles R. Drew University of Medicine and Science, Los Angeles, CA, United States; 4 Marginalization-Related Diminished Returns (MDRs) Center, Los Angeles, CA, United States; 5 Department of Health Policy and Management, Johns Hopkins Bloomberg School of Public Health, Baltimore, MD, United States; 6 School of Business, University of Maryland Global Campus (UMGC), College Park, MD, United States

**Keywords:** Racism, Discrimination, Racial Beliefs, Parental Education, Ethnic Groups

## Abstract

**Background:**

Racial discrimination remains a significant issue in the United States, particularly affecting Black individuals. Understanding how beliefs about the persistence of racial discrimination are shaped by race and parental education among college students is crucial for developing strategies to address these inequities.

**Objectives:**

This study aims to examine the multiplicative effects of race and parental education on beliefs about the persistence of racial discrimination among Black and White college students. We hypothesize that Black students, particularly those with highly educated parents, will exhibit stronger beliefs in the persistence of racial discrimination as a significant issue compared to their White peers.

**Methods:**

Data were drawn from the Higher Education Research Institute (HERI) study, which includes a national sample of Black and White young adults on college campuses across the United States. We conducted statistical analyses to explore the influence of race and parental education on students’ beliefs about racial discrimination.

**Results:**

Black students demonstrated stronger beliefs in the persistence of racial discrimination compared to White students. Additionally, students with higher parental education levels were more likely to recognize racial discrimination as a significant issue than those with lower parental education. The impact of parental education on these beliefs was more pronounced for Black students compared to White students.

**Discussion:**

Black college students, especially those from higher SES backgrounds, exhibit a heightened awareness of racial discrimination due to their lived experiences and educational exposure. High SES Black individuals often face increased visibility and prejudice in predominantly White environments, further reinforcing their beliefs about the persistence of discrimination. These findings highlight the need for targeted interventions to support Black students in higher education and promote awareness of racial discrimination. Policy solutions should include comprehensive anti-discrimination policies, increased diversity and inclusion efforts, and educational curricula that address systemic racism and social justice. The cross-sectional nature of the data limits causality inference, and self-reported measures may be subject to bias. Despite these limitations, the study’s large and diverse sample enhances the generalizability of the findings. Race and parental education have multiplicative effects on college students’ beliefs about the persistence of racial discrimination. Black students, particularly those from high SES backgrounds, are more likely to perceive racial discrimination as a continuing problem. Addressing these disparities through targeted policies and interventions is essential for creating equitable and inclusive educational environments.

## 1. Introduction

Racial discrimination remains a pervasive issue in the United States, with Black individuals facing systemic inequities across various domains, including education, employment, and healthcare [1–5]. Despite legislative advancements and societal progress, racial discrimination continues to manifest in both overt and covert forms, significantly impacting the lives and opportunities of Black people [6–8]. This persistent problem necessitates ongoing examination and intervention to address and dismantle the structures that uphold racial inequities [2,9–13].

Public perceptions of racial discrimination vary widely [14,15], often influenced by individual experiences, social environment, and educational background [16–18]. Many Americans recognize racial discrimination as a critical issue, with differing degrees of acknowledgment and concern across racial and socioeconomic lines [19,20]. These beliefs and attitudes towards the persistence of racial discrimination are crucial in shaping public discourse, policy-making, and collective efforts to promote racial equity [21].

Educational attainment and racial identity significantly influence individuals’ beliefs about the persistence of racial discrimination. Higher levels of education often correlate with a greater awareness and acknowledgment of systemic inequalities [19]. Moreover, Black individuals, particularly those with higher education levels, are more likely to perceive racial discrimination as a sustained and continuing problem [22,23]. This intersection of race and education creates a complex landscape where beliefs about racial discrimination are shaped by both individual and contextual factors [24–30].

Recent studies on Minorities’ Diminished Returns (MDRs) indicate that Black individuals with high socioeconomic status (SES) experience greater levels of discrimination compared to their White counterparts [31]. This phenomenon suggests that the protective effects of SES are less potent for Black individuals, leading to higher exposure to discrimination despite their achievements [32]. Consequently, the combined effects of race and parental education on beliefs about racial discrimination are likely to be multiplicative, with Black college students from highly educated families being more attuned to and affected by these issues [33–35].

## 2. Aims and Hypothesis

This study aims to examine the multiplicative effects of race and parental education on beliefs about racial discrimination among Black and White college students in the United States. Using data from the Higher Education Research Institute (HERI) study, which includes a national sample of young adults on college campuses, we hypothesize that Black students, particularly those with highly educated parents, will exhibit stronger beliefs in the persistence of racial discrimination as a significant issue compared to their White peers. By exploring these dynamics, we seek to contribute to a deeper understanding of the interplay between race, education, and perceptions of racial discrimination, ultimately informing strategies to address and mitigate racial inequities in society.

By exploring these dynamics, we seek to contribute to a deeper understanding of the interplay between race, education, and perceptions of racial discrimination, ultimately informing strategies to address and mitigate racial inequities in society.

## 3. Methods

### 3.1. Design and Setting

This study is cross-sectional and utilizes data from the American Freshman surveys spanning from 1966 to 2019. The American Freshman surveys, conducted annually, capture over 40 years of insights into the characteristics, attitudes, values, educational achievements, and aspirations of college students in the United States. Initiated by the Cooperative Institutional Research Program (CIRP) every fall since 1966, these surveys provide a comprehensive view of the evolving nature of American college students.

These annual surveys reflect not only changes within higher education but also broader societal shifts. This report offers an overview of the first twenty-five years of CIRP data, highlighting significant findings and their implications for both American higher education and society. The initial seven surveys were conducted by the American Council on Education, with funding from the Carnegie Corporation of New York and the Ford Foundation. Since 1972, the Higher Education Research Institute (HERI) at the University of California, Los Angeles, has taken over the annual CIRP freshman surveys, with continued support from the American Council on Education.

Each year, CIRP surveys approximately 250,000 full-time students from around 600 two- and four-year colleges and universities across the nation. The data presented in this report are categorized into eight broad topics: academic skills and preparation, demographic trends, high school activities and experiences, educational and career plans, majors and careers, attitudes, student values, and financing college.

### 3.2. Measures

Variables in the current analysis were race, ethnicity, sex, survey year, parental education, and belief/attitude about discrimination. Race, ethnicity, sex, parental education, and belief/attitude about discrimination were all self-reported. Race was Black and White. Ethnicity was Latino vs. Non-Latino. Age group was an ordinal variable ranging from 1 to 11. 1: 16 or younger; 2: 17; 3: 18; 4: 19; 5: 20; 6: 21 or older; 7: 22 to 24; 8: 25 to 29; 9: 30 to 39; and 10: 40 to 54. Parental education was an ordinal variable ranging from 1 to 8. 1: Junior High; 2: Some High School; 3: High School Graduate; 4: Post Secondary School Other than College; 5: Some College; 6: College Degree; 7: Some Graduate; and 8: Graduate Degree. Male was coded 1 and female was coded 0 for sex.

### 3.3. Statistical Analysis

For the descriptive analysis, we calculated frequencies and percentages to summarize the distribution of the key variables, including race, parental education, belief in discrimination, age, sex, and year of survey. Bivariate associations between these variables were assessed using chi-square tests for categorical variables and t-tests for continuous variables.

For the multivariable analysis, we conducted logistic regression models to examine the relationship between parental education and belief in discrimination, with race as a moderator and controlling for confounders such as age, sex, and year of survey.

- **Model 1** included the main effects of parental education and race, along with the confounders.
- **Model 2** added an interaction term between race and parental education to test whether the effect of parental education on belief in discrimination varied by race.

Both models reported odds ratios (ORs) and 95% confidence intervals (CIs) to quantify the strength and precision of the associations. All statistical analyses were performed using appropriate software, and a significance level of 0.05 was used to determine statistical significance.

## 4. Results

Overall, a total number of 1,464,839 participants entered our analysis. Table 1 shows summary of descriptive data.

Table 2 shows he summary of logistic regression without interaction terms in the model. As this Table shows, high parental education and being Black were associated with higher odds of believing that discrimination is still a problem in the US.

Table 3 shows he summary of logistic regression with an interaction term between race and parental education in the model. As this Table shows, the positive association between high parental education and the odds of believing that discrimination is still a problem in the US was larger (stronger) for Black than White students.

## 5. Discussion

Our findings reveal that Black students demonstrate stronger beliefs in the persistence of racial discrimination compared to their White counterparts. Additionally, young adults with higher parental education levels are more likely to recognize racial discrimination as a significant issue than those with lower parental education. However, the impact of parental education on these beliefs differs by race, with Black students showing a more pronounced positive effect compared to White students. This suggests that the interplay between race and socioeconomic status shapes perceptions of racial discrimination in complex ways.

Racial discrimination remains a pervasive issue in the United States, deeply rooted in the nation’s history and structural inequalities [36–40]. Despite significant legal and social advancements, discriminatory practices persist in various domains such as housing, employment, education, and the criminal justice system [41]. The enduring nature of racial discrimination is fueled by systemic racism, implicit biases, and institutional practices that disproportionately disadvantage Black individuals [2,5,42,43]. These ongoing challenges highlight the need for continuous efforts to address and dismantle the structures that sustain racial inequities.

Race significantly influences individuals’ perspectives on racial discrimination, with Black college students more likely to perceive discrimination as an ongoing problem [44–46]. This heightened awareness among Black students can be attributed to their lived experiences and the societal challenges they face due to their racial identity. Education also plays a crucial role in shaping these perspectives, as higher educational attainment often leads to a greater understanding of systemic inequalities [47–50]. Mechanisms such as increased exposure to diverse viewpoints, critical thinking skills, and access to information about social justice issues contribute to this heightened awareness [51]. For Black students, education not only enhances their understanding of racial discrimination but also reinforces their recognition of its persistence in society [52,53].

Similarly, parental education influences beliefs about racial discrimination, with young adults from higher socioeconomic backgrounds more likely to recognize it as a significant issue. However, this effect is more pronounced in Black families [19]. High SES Black individuals often find themselves in predominantly White environments where they may experience heightened visibility and, consequently, greater exposure to racial prejudice [54,55]. This increased exposure can amplify their awareness of racial discrimination and reinforce their beliefs about its persistence. Mechanisms such as social comparison, experiences of microaggressions, and heightened scrutiny in these environments contribute to this phenomenon [22].

### 5.1. Implications

The findings of this study have important implications for understanding the experiences of college students and the upward mobility of Black students. Recognizing the persistence of racial discrimination is crucial for developing strategies to support Black students in higher education. Institutions must acknowledge the unique challenges faced by Black students and implement policies that foster inclusive and supportive environments [56–59]. Addressing the differential impact of parental education on beliefs about racial discrimination can also inform targeted interventions to promote awareness and resilience among students from diverse backgrounds.

### 5.2. Policy Solutions

To address the persistence of racial discrimination and support the upward mobility of Black students [60,61], several policy solutions can be considered. These include implementing comprehensive anti-discrimination policies, increasing diversity and inclusion efforts on college campuses, and providing targeted support services for Black students. Additionally, educational curricula should incorporate critical discussions on systemic racism and social justice to enhance students’ understanding of these issues [22]. Policy makers must also focus on addressing broader structural inequalities that contribute to racial discrimination, such as disparities in housing, employment, and criminal justice [4,62–69].

### 5.3. Limitations and Strengths

This study has several limitations that should be acknowledged. First, the cross-sectional nature of the HERI data limits our ability to infer causality. Second, self-reported measures of beliefs about racial discrimination may be subject to social desirability bias. Future research may be on implicit bias [70]. Despite these limitations, the study’s strengths include its large and diverse national sample, which enhances the generalizability of the findings. Additionally, the focus on multiplicative effects of race and parental education provides a nuanced understanding of the factors influencing beliefs about racial discrimination among college students.

## 6. Conclusions

In conclusion, this study highlights the complex and non-linear effects of race and parental education on beliefs about the persistence of racial discrimination among college students. Black students, particularly those from higher parental education backgrounds, exhibit stronger beliefs in the persistence of racial discrimination compared to their White peers. These findings underscore the importance of addressing racial discrimination and promoting awareness and resilience among college students. By implementing targeted policies and interventions, institutions and policymakers can work towards creating more equitable and inclusive environments that support the success and well-being of all students.

## Figures and Tables

**Table 1.** Descriptive Characteristics

	N	%
**Race**		
White	1427059	97.4
Black	37780	2.6
**Sex**		
Female	852632	58.2
Male	612207	41.8
**Parent Education**		
1: Junior High	82387	5.6
2: Some High School	87842	6.0
3: High School Graduate	228910	15.6
4: Post Secondary School Other than College	50727	3.5
5: Some College	240339	16.4
6: College Degree	365004	24.9
7: Some Graduate	40455	2.8
8: Graduate Degree	369175	25.2
**Age (Years)**		
16 or younger	2074	0.1
17	50411	3.4
18	969053	66.2
19	309060	21.1
20	50903	3.5
21 or older	52763	3.6
22 to 24	15451	1.1
25 to 29	10743	0.7
30 to 39	3942	0.3
40 to 54	439	0.0
**Survey Year**		
1989	4173	0.3
1990	46814	3.2
1991	52916	3.6
1992	53609	3.7
1993	54804	3.7
1994	62191	4.2
1995	58718	4.0
1996	65224	4.5
1997	66216	4.5
1998	73835	5.0
1999	76173	5.2
2000	87624	6.0
2001	90826	6.2
2002	89845	6.1
2003	86812	5.9
2004	93778	6.4
2005	82203	5.6
2006	84905	5.8
2007	83152	5.7
2008	80470	5.5
2009	70551	4.8
**Discrimination Still a Problem**		
No	207235	14.1
Yes	1257604	85.9

**Table 2.** Summary of Model 1 (Logistic Regression)

	B	S.E.	Exp(B)	95% C.I.for EXP(B)		Sig.
Male (1)	−.458	.005	.632	.626	.638	< .001
Age (1_11)	−.089	.002	.915	.912	.918	< .001
Survey Year	−.021	.000	.979	.978	.980	< .001
Race (Black)	.226	.016	1.254	1.214	1.295	< .001
Parent education (1–8)	.038	.001	1.039	1.037	1.041	< .001

**Table 3.** Summary of Model 2 (Logistic Regression)

	B	S.E.	Exp(B)	95% C.I.for EXP(B)		Sig.
Male(1)	−.458	.005	.632	.627	.638	< .001
Age (1_11)	−.089	.002	.915	.912	.918	< .001
Survey Year	−.021	.000	.979	.978	.980	< .001
Race (Black)	−.056	.050	.946	.857	1.044	< .001
Parent education (1–8)	.037	.001	1.038	1.036	1.040	< .001
Parent education (1–8) x Black	.050	.009	1.051	1.034	1.069	< .001

## Data Availability

HERI data are available to public at https://heri.ucla.edu/.
